# Retroaortic left innominate vein – Incidence, association with congenital heart defects, embryology, and clinical significance

**DOI:** 10.4103/0974-2069.43881

**Published:** 2008

**Authors:** Snehal Kulkarni, Shreepal Jain, Pankaj Kasar, Swati Garekar, Suresh Joshi

**Affiliations:** Pediatric and Congenital Heart Center, Wockhardt Hospital, Mumbai, India

**Keywords:** Retroaortic left innominate vein, right aortic arch, tetralogy of Fallot

## Abstract

In a retrospective analysis of echocardiograms, the incidence of retroaortic innominate vein was found to be 0.55% amongst children with congenital heart disease. It was most commonly associated with tetralogy of Fallot and right aortic arch.

## INTRODUCTION

The left innominate (or brachiocephalic) vein is formed by the left internal jugular and the left subclavian vein. In the situs solitus, its usual course is obliquely downward to the right, passing superoanterior to the aortic arch. Anomalous course of the innominate vein is rare, being first described by Kershner more than 100-years ago.[1] Incidence of this anomaly is reported to be between 0.2–1% of all congenital cardiac defects.[2–4]

More than 80% of the patients with anomalous left innominate vein have obstruction of the right ventricular outflow tract, commonly tetralogy of Fallot (TOF) with or without pulmonary atresia.[5,6] Right aortic arch is a common association.[7] It is occasionally seen with total anomalous pulmonary venous return.[6] Anomalous retroaortic course of the left innominate vein can be diagnosed on routine transthoracic two-dimensional echocardiography (TTE) with color flow imaging.

The present study was conducted to evaluate the association of retroaortic left innominate vein with various congenital heart defects and describe its clinical significance.

## MATERIALS AND METHODS

**Figure 1:**
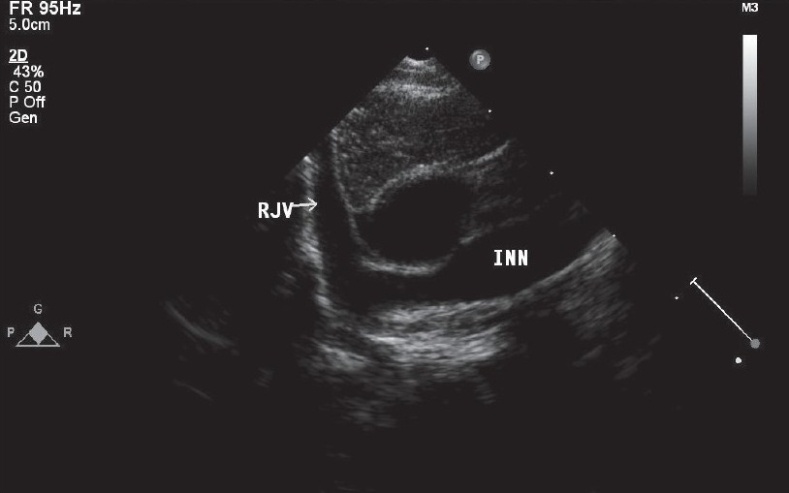
Suprasternal short axis view showing the retroaortic course of the left innominate vein

We analyzed the echocardiograms of all patients between February 2007 and June 2008 to assess the types of cardiac defects and the number of patients with retroaortic course of innominate vein. Retroaortic innominate vein was diagnosed on routine TTE with color flow mapping using suprasternal short axis view [Figure 1]. In this view, the left internal jugular vein was seen coming below the aortic arch and spectral Doppler imaging confirmed low velocity venous flow. CT scan or MRI was not done in any of them. The presence of retroaortic left innominate vein was confirmed during cardiac surgery in all operated patients. Chi square test was used for statistical analysis and a *P* value of <0.05 was considered as significant.

## RESULTS

Of 1600 echocardiograms performed during the study period, 168 echocardiograms (10.5%) turned out to be normal with none having a retroaortic left innominate vein. A total of eight patients out of the remaining 1432 (0.55%) were detected to have a retroaortic innominate vein. Seven among these had TOF and one patient had mitral atresia, double outlet right ventricle with a ventricular septal defect, and severe pulmonary stenosis. Of the seven patients with TOF who had retroaortic innominate vein, two had pulmonary atresia and six patients had right-sided aortic arch. Among the 1432 patients analyzed, 82 patients (5.7%) were detected to have TOF. Thus, seven out of 82 (8.5%) patients with TOF had a retroaortic innominate vein. Retroaortic innominate vein was more common in those with right aortic arch (6 out of 15) than with left aortic arch (1 out of 67); (*P* < 0.01). The incidence of retroaortic innominate vein was not significantly different in patients with TOF (5 out of 66) as compared to those with TOF and pulmonary atresia (2 out of 16); (*P* = NS) [Table 1].

**Table 1:** Data of the patients with retroaortic left innominate vein

Patient sr. no.	Age/sex (years) (M/F)	Diagnosis	Right aortic arch	Pulmonary atresia
1	0.4/ M	Mitral atresia, hypoplastic LV, DORV, VSD, PS	No	No
2	0.8/F	TOF	Yes	No
3	1.5/M	TOF	Yes	No
4	16/F	TOF	Yes	Yes
5	1.5/M	TOF, PA, post BT shunt	No	Yes
6	1.5/M	TOF	Yes	No
7	0.4/F	TOF, severe PS, post BT shunt	Yes	No
8	0.1/M	TOF, severe PS	Yes	No

TOF: Tetralogy of Fallot; DORV: Double outlet right ventricle; PS: Pulmonic stenosis; PA: Pulmonary atresia

## DISCUSSION

Our study showed 0.55% incidence of retroaortic innominate vein among patients with congenital heart defect. It had commonest association with TOF as has been described previously by others.[3,5–7] In addition, it was more common in patients with TOF associated with right aortic arch. The association of retroaortic innominate vein with TOF and pulmonary atresia was not statistically significant as compared to those with TOF without pulmonary atresia in the present study. It was correctly diagnosed in those patients who underwent surgical repair, indicating a high sensitivity of TTE in diagnosing this anomaly.

Embryologically, the primordia of the systemic veins first appear as paired anterior and posterior cardinal veins that unite on each side to form a common cardinal vein (or Cuvierian duct) that opens into the primitive sinus venosus. During subsequent development, most of the left anterior cardinal vein disappears. The venous drainage from the left side of the head and neck and the left arm is then directed into the right anterior cardinal vein by the development of new transverse anastomotic channels above and below the fourth aortic arch (superior and inferior transverse capillary plexus) by the eighth week. Normally, the aortic arch shortens during the embryological development and occupies the space of the inferior transverse capillary plexus, thus causing its regression, while the rest of venous blood shunts into the superior transverse capillary plexus. This facilitates the development of the normal supra aortic course of the left innominate vein.

As against this, reduced shortening of the aortic arch as seen in right aortic arch and high aortic arch may compress and prevent the further development of the superior transverse venous plexus. Abnormal development of the pulmonary arteries, either pulmonary atresia or pulmonary stenosis, encourages the sparing of the inferior transverse plexus, possibly leading to formation of an anomalous course of the innominate vein. This would explain frequent association of the retroaortic innominate vein with TOF and right aortic arch.[3,8,9]

Usually, the retroaortic innominate vein in isolation has no clinical importance. The descending portion of the retroaortic innominate vein may be mistaken for persistent left superior vena cava or an ascending vertical vein in a total anomalous pulmonary venous connection on echocardiography.[3,10] The retroaortic segment may be misinterpreted as right pulmonary artery in patients with hypoplastic or atretic central pulmonary arteries, or an early branching of right upper lobe pulmonary artery on echocardiography.[2,4,7]

The anomalous innominate vein may cause technical difficulties during pacemaker insertion or central venous line placement through the left arm approach. For patients undergoing cardiac surgery, the superior vena caval cannulation for cardiopulmonary bypass has to be done more caudally than usual to avoid obstruction of the retroaortic innominate vein.[7] The anomaly may complicate exposure of the pulmonary arteries while creating systemic vein to pulmonary artery anastomosis during Glenn shunt. Also, it may obscure the surgical field in the construction of a subclavian to pulmonary artery shunt and the ligation of a patent ductus arteriosus.[3,11] Precise preoperative information about this anomaly can be critical in planning the surgical procedure. Agarwal *et al*, reported a case of tricuspid atresia and pulmonary atresia with bifurcation stenosis of confluent pulmonary arteries where the retroaortic vein was used for pulmonary artery reconstruction while performing a cavopulmonary anastomosis.[12] Baba *et al*, reported another case with superoinferior ventricles with retroaortic innominate vein where it was used for a right atrial pulmonary anastomosis.[13]

To conclude, though presence of retroaortic innominate by itself may be of no clinical significance, it should be looked for carefully, especially in patients with right ventricular outflow tract obstruction, to decide the plan during surgery or interventions. The information may also be useful in certain cases requiring unconventional surgical repairs.
